# Will open access increase journal CiteScores? An empirical investigation over multiple disciplines

**DOI:** 10.1371/journal.pone.0201885

**Published:** 2018-08-30

**Authors:** Yang Li, Chaojiang Wu, Erjia Yan, Kai Li

**Affiliations:** 1 LeBow College of Business, Drexel University, Philadelphia, Pennsylvania, United States of America; 2 College of Computing and Informatics, Drexel University, Philadelphia, Pennsylvania, United States of America; Peking University First Hospital, CHINA

## Abstract

This paper empirically studies the effect of Open Access on journal CiteScores. We have found that the general effect is positive but not uniform across different types of journals. In particular, we investigate two types of heterogeneous treatment effect: (1) the differential treatment effect among journals grouped by academic field, publisher, and tier; and (2) differential treatment effects of Open Access as a function of propensity to be treated. The results are robust to a number of sensitivity checks and falsification tests. Our findings shed new light on Open Access effect on journals and can help stakeholders of journals in the decision of adopting the Open Access policy.

## Introduction

The notion of open science is as old as Scientific Revolution. During the 16^th^ and 17^th^ centuries, science began to diverge from the paradigm of “secrecy in the pursuit of nature’s secrets” [1] that had predominated in the Middle Ages. Openness is inscribed in the modern scientific norms summarized by Robert Merton [2]. This value was embodied in the broad open access movement, which originated in the early 1990s with the establishment of the first open access journals [3] and arXiv.org [4, 5]. The movement came into focus with the release of the Budapest Open Access Initiative (BOAI), the first international and open public statement concerning open access principles [6]. BOAI was later accompanied by the Bethesda Statement on Open Access Publishing [7], the Berlin Declaration on Open Access to Knowledge in the Sciences and Humanities [8], and other sister initiatives as well as local policies.

Two approaches to open access were defined in BOAI: gold open access (open access publications available directly from the publisher) and green open access (publications available through self-archiving by the authors) [9–11]. Within each of these broad approaches, there are different modes of implementation. For example, three types of gold open access have been identified: Direct OA (the entire journal is published as open access), Delayed OA (the latest contents are only available to paid users), and Hybrid OA (authors pay to a subscription-based journal for the content to be open access) [12].

As the idea of open access has been increasingly grounded, the popularity of open access journals has increased drastically during the past two decades, as documented in various empirical studies. One stream of such studies has measured the numbers of open access papers, journals, repositories, and publishers [9, 13, 14]. Though clearly increasing over time, these numbers are only one indicator of the impact of open access publications.

This study intends to assess the impact of open access journals from the perspective of citation advantage. Prior studies of this topic have typically compared the impact difference between closed access and open access journals on the level of either papers or journals. Even though most studies seem to support the citation advantage of open access journals or articles [12, 15], many have limited coverage of knowledge domains and are thus insufficient to draw conclusions concerning cross-disciplinary differences in impact. In this study, we have included a comprehensive list of open access journals representing a wide range of knowledge domains. Our unique dataset contains a large pool of non-OA journals which enable us to find close controls for the OA journals for better causal inferences.

Furthermore, taking advantage of the controls, we employ a difference-in-difference identification strategy intended to identify and measure the causal effect of OA–a strategy rarely used in previous studies of open access, which have approached the effect of open access in a more descriptive way, by simply comparing the OA journals with non-OA journals. Methods used in these prior studies include descriptive statistics [16–18], simple statistical inference such as t-test or its variants [19, 20], and a regression-based method with control variables [21, 22]. All these methods, however, suffer from a common issue: namely, the conclusions are drawn without a proper control group. That is, the OA articles/journals and non-OA articles/journals are different and may not be comparable. It may well be that higher quality articles/journals elect to adopt OA, in which case the subsequent citation advantage could be due either to OA or to the higher quality of the publications. One study which did confront this issue is that of Davis et al. [23], who employed randomized controls; however, the randomized controls experiment can be quite expensive, and in most scenarios this approach is not applicable. In this study, we are able to find suitable controls for the OA journals within the same field and with similar standings. Thus, we can draw a causal inference regarding the effect of OA.

In addition, we investigate two sources of heterogeneous treatment effect: (1) the differential treatment effect among journals grouped by academic field, publisher, and tier; and (2) differential treatment effects of Open Access as a function of propensity to be treated. Our study of heterogeneous effect of OA contributes to the literature regarding “long tail” vs. “superstar” effect in the journals market. On one hand, similar to the “long tail” effect [24] in retail markets that sales of obscure or niche products might benefit from internet search and acquisition, low-ranked journals may boost their cites when the full text of articles are available online through internet search. Particularly, the effect of OA is likely to be marginal for “dominant” journals that are already widely cited before open access. On the other hand, as argued by McCabe and Snyder [22], “superstar” effect may at work by intensifying competition among journal articles after OA. That is, highly ranked journals will be cited even more because of their quality. We argue that obscure journals might benefit from online availability facilitated by OA only if they are quality journals that have potential in the growth of their CiteScores. Using pre-OA CiteScore to proxy for journal quality and journal tier for the popularity of a journal, our results of differential treatment effects based on propensity suggest that the quality obscure journals, in the sense of having higher pre-OA CiteScores and lower ranks, are most likely to open access and thus benefit more from becoming open access.

## Literature review

As open access becomes a central topic in scholarly communication, it has also gained more attention from scientometrics researchers, who have compared the citation rates between OA and non-OA publications in various contexts. Summarizing these empirical studies, Swan [25] and Tennant et al. [26] found a general consensus that the impact of a paper is increased if it is published as open access. Key parameters in determining this effect include the level of research object (journals vs. articles) and the knowledge domains in which the research is located.

Overall, there seems to be widespread agreement that OA can increase the citation of a study in a controlled situation [9, 15, 19, 20, 27–29]. In the published research on this topic to date, knowledge domain is arguably the most important factor in determining the outcome. For instance, a longitudinal study conducted by Hajjem et al. [29] demonstrated that OA articles are 36% to 172% more likely to be cited than their non-OA counterparts across a broad array of knowledge domains. Similarly, Antelman [19] showed that citation rates for OA publications exceed those for non-OA publications by 91%, 51%, 86%, and 45% in mathematics, electrical engineering, political science, and philosophy, respectively. Xu, Liu, and Fang [30] observed that, in contrast to all other knowledge domains, OA papers in the humanities are less cited than non-OA papers.

What should be noted, however, is that these studies have adopted highly varied methods in their analysis. Their choice of metrics, including altmetrics, has varied greatly, as have their efforts to account for selection bias and the early view effect. As mentioned above, these differences in research design affect the translatability of their findings; moreover, they often lead to disparate conclusions. Most previous studies have used the number of citations as the single indicator of the impact of papers, but a significant minority [18, 31–33] have employed altmetrics, including but not limited to the number of downloads and web page visitors. Only a few studies to date have used a transformed index, more standardized than citation count, to represent the impact of publications [22, 34]. By using one such index, CiteScore, the present study aims to enrich our understanding of the measures of a work’s scholarly impact. We will discuss the CiteScore measure in the next section.

Furthermore, previous studies report ambiguous findings of the effect of OA across journals of different ranks. For example, Evans [35] reports that as more journals come online, the citation of articles tends to concentrate on fewer journals and articles, suggesting a “superstar” effect. McCabe and Snyder [22] also find a “superstar” effect of OA on citations using panel data of science journals, they find that top-50% journals benefitted more than bottom-50% journals from OA. McCabe and Snyder [36], however, find that the effect of OA is fairly uniform across the rank of cited journals, supporting both “long tail” and “superstar” effect. These controversial results make further investigation on heterogeneity in the effect of OA necessary in this field.

## Data and descriptive statistics

Two data sources were used in this study: Journal Metrics by Scopus (https://journalmetrics.scopus.com/) and the Directory of Open Access Journals (DOAJ). We used Journal Metrics to obtain journal bibliometric data including CiteScore, quartiles, and subject areas and DOAJ to obtain a journal’s open access information. Note that the OA journals included here are direct open access journals, which means the entire journal became open access during our observation period. We do include the journals that contain open access articles elected by the authors. We conduct additional analyses and find no significant impact to our results. Details are discussed in the robustness check section.

CiteScore was proposed by Elsevier in 2016 as a measure of journals’ citation impact. In contrast to journal impact factor, CiteScore uses a three-year citation window and includes all document types in its calculation. Our choice of CiteScore as a proxy for scientific impact is in line with previous research [12], though we are aware of the concerns inherent in relying on a single indicator in conducting research evaluations [37]. It is beyond the scope of this study to discuss the limitations of CiteScore-like indicators.

In ranking journals on the basis of their CiteScores, Scopus uses four quartiles plus a fifth category called “Top 10%”, which includes journals in the 99th to 90th percentile; thus, in effect, Quartile 1 includes journals between the 89th and 75th percentile. However, Quartile 1 includes Top 10% journals in the searchable database. In addition, Scopus assigns journals into 27 major subject areas and 334 minor subject areas. For ease of presentation, we further merged the focal major subject areas into six broad domains: Biology, Engineering, Math & Computer science, Medicine, Science, and Social science. Table 1 presents our categorization scheme of major subject areas. Note that we drop the major subject area “Multidisciplinary” from our categorization scheme because there is no multidisciplinary journal in our data. We designate Springer, Sage, Elsevier, Wiley-Blackwell, and Taylor & Francis as the “Big Five” publishers [38], since they are the institutions with established reputations.

**Table 1:** Merging 26 major subject areas into 6 broad domains.

Major subject areas	Merged domains
Biochemistry, genetics and molecular biology Immunology and microbiology	Biology
Chemical engineering Energy Engineering	Engineering
Computer science Decision sciences Mathematics	Math & Computer science
Dentistry Health professions Medicine Nursing Pharmacology, toxicology and pharmaceutics Veterinary	Medicine
Agricultural and biological sciences Chemistry Earth and planetary sciences Environmental science Materials sciences Neuroscience Physics and astronomy	Science
Arts and humanities Business, management and accounting Economics, econometrics and finance Psychology Social sciences	Social science

To accurately determine the year that a journal moved from closed access to open access, we cross-referenced Journal Metrics data with data from DOAJ that specifies the year in which the change took place. In total, 244 instances of journals moved from closed access to open access between 2011 and 2014. (“Instance” here means a journal and its assigned domain—the two uniquely identify one instance.) These instances form the treatment group in this study. For each journal instance in the treatment group, its control group includes journals that are in the same minor subject and the same rank category within that subject. On average, each journal instance in the treatment group has about 60 journals in the control group.

Table 2 presents mean CiteScore and frequencies for the main categorical variables in our sample of journal-year observations. Column 1 reports data for the full sample; columns 2 and 3 report data for OA journals (treatment group) and non-OA journals (control group) respectively. There are a total of 66,135 observations in the full sample with an average CiteScore of 0.886. As Table 2 shows, OA journals, compared to non-OA journals, have higher CiteScore on average: 1.362 for the former versus 0.877 for the latter. The frequencies for categorical variables for OA journals, such as publisher, tier, and domain, are commensurate with those for non-OA journals.

**Table 2:** Mean and frequencies of main variables.

Variable	All journals (1)	OA journals (2)	Non-OA journals (3)
CiteScores	0.886	1.362	0.877
By publisher			
Big five publishers	21,155	390	20,765
Other publishers	44,980	830	44,150
By tier			
Top 10%	2,020	90	1,930
Quartile 1	5,275	205	5,070
Quartile 2	19,015	330	18,685
Quartile 3	21,135	390	20,745
Quartile 4	20,710	295	20,415
By domain			
Biology	8,935	245	8,690
Engineering	6,115	125	5,990
Math & CS	3,425	60	3,365
Medicine	18,195	265	17,930
Science	13,965	280	13,685
Social science	15,500	245	15,255
Total observations	66,135	1,220	64,915

**Fig 1:**
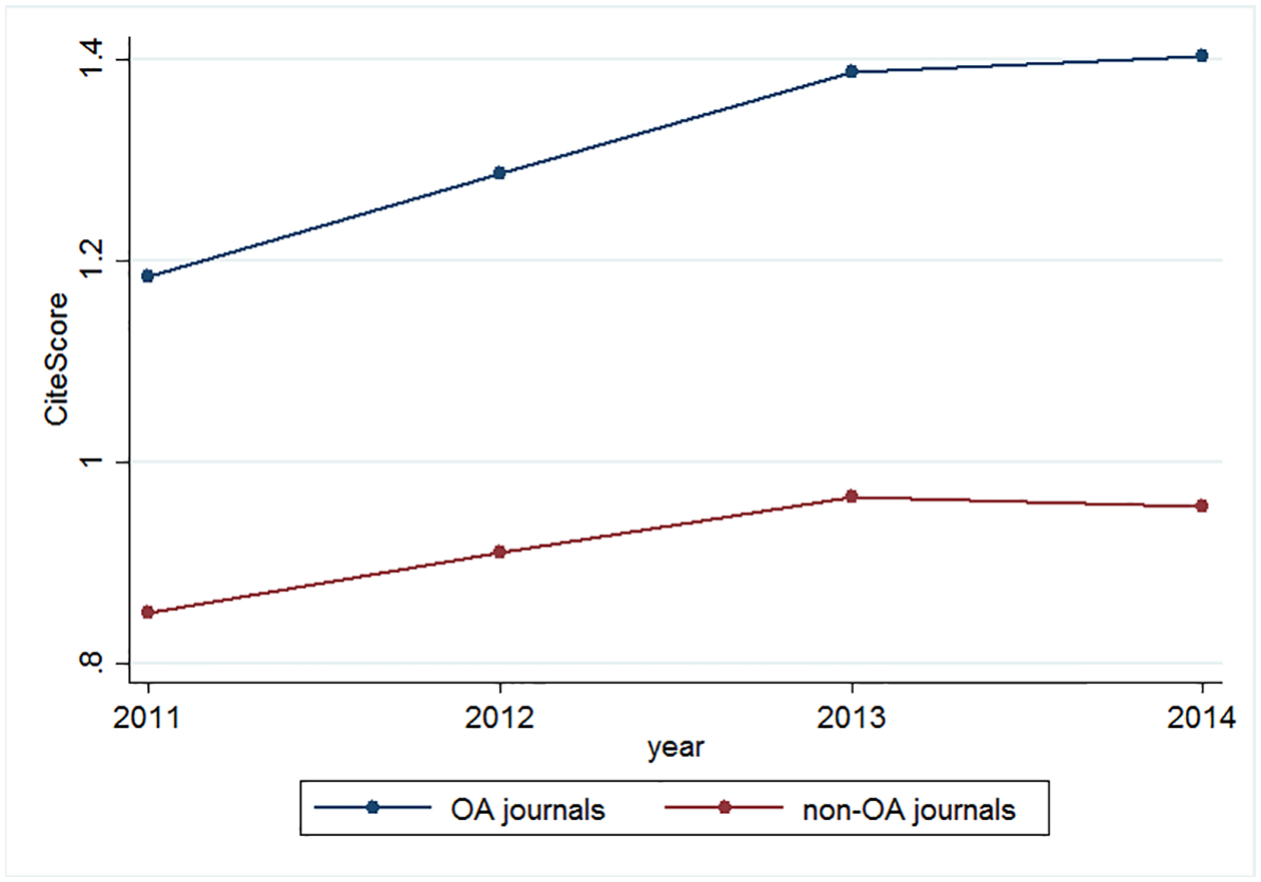
CiteScores by year.

We also summarize the mean CiteScores by year from 2011-2014 in Fig 1. The change in mean CiteScore for OA journals during this period is similar to that for non-OA journals. All in all, there is no obvious difference between the treatment and control groups in terms of either journal characteristics or the trend in CiteScores.

## Statistical methodology

We first examine the effect of Open Access (OA) on journal CiteScores using difference-in-difference [39, 40]. Given the panel structure of our data, we use difference-in-difference to derive an unbiased estimator of the effect of OA on CiteScore. We further conduct a sample-splitting test [41, 42] in an attempt to test the heterogeneous treatment effects of OA on CiteScores over subsamples of data based on journal characteristics. Moreover, we adopt the stratification-multilevel method [43] to analyze the heterogeneous effects of OA as a function of the likelihood of OA.

### Difference-in-difference

The difference-in-difference method evaluates the treatment effect by comparing the change in the outcome of interest before and after the intervention for both treatment and control groups. The method eliminates selection bias by subtracting the pre-treatment level of the outcome from the post-treatment level. By assuming a parallel trend [44] between treatment and control group, the difference-in-difference delivers an unbiased estimate of the effect of a change in access policy. Moreover, the parallel-trend assumption allows the difference-in-difference to account for time-invariant unobserved variables in a panel data setting [45].

The validity of a difference-in-difference estimate depends on the degree of similarity between the treatment and control groups: if the trends between the two groups were significantly different, the assumption of a parallel trend would be violated. Another key assumption, the common shocks assumption [46], states that apart from the treatment itself, any other events occurring during or after the time the treatment is given will equally affect the treatment and control groups. This means the only difference between two groups in outcome variable would be exposure to the treatment. Therefore, the main difficulty in implementing difference-in-difference method for empirical studies lies in finding treatment and control groups sufficiently similar to satisfy these assumptions. In this study, we alleviate this concern by building our control group in such a way that non-OA journals are matched to the most similar OA journals based on discipline and tier.

In our dataset, although the CiteScore and open access events are observed during the years 2011-2015, we only consider shifts to OA during the years 2011-2014. In this way, at least one year of potential impact can always be observed. By regarding treatment in each year as a distinct event, we arrive at four treatment events to be evaluated in our study. For each event, we estimate the effect of OA on CiteScore using the following specification:

$$\begin{array}{c} y_{it}=\delta R_{it}+\beta trend+\lambda_{t}+\alpha_{i}+\mu_{it} \end{array}$$ (1)

In the above formula, *y*_*it*_ is the CiteScore for journal *i* at year *t*; λ_*t*_ and *α*_*i*_ are year and journal fixed effects; *R*_*it*_ is a dummy variable that equals 1 if journal *i* becomes open access by year *t* − 1 and 0 otherwise; and *μ*_*it*_ is an error term. We also include a linear time trend in regression to account for the growth of CiteScore over years. For the event in each year, our estimate of the treatment effect is *δ*. Eq 1 uses fixed effects because they can control for the unobserved differences across journal and time, while at the same time serving as treatment or post dummies [47].

Apart from investigating the effects of OA as separate events occurring in different years, we study the overall treatment effect by addressing the following question: how might multiple OA treatment as a whole, albeit across different years, influence the CiteScore? To answer that question, we follow Gormley and Masta [48] and use fixed effects to control for the unobserved differences arising from journals, years, and events. To be specific, we estimate:

$$\begin{array}{c} y_{ict}=\delta R_{ict}+\beta trend+\lambda_{ct}+\omega_{ic}+\mu_{ict} \end{array}$$ (2)

where *y*_*ict*_ is the CiteScore for journal *i*, treated in cohort *c* (an index representing the year in which the OA event took place), in year *t*; *R*_*ict*_ is a dummy variable that equals 1 if journal *i* becomes open access by year *t* − 1 in cohort *c* and 0 otherwise; *trend* is a linear trend that accounts for the growth of CiteScore; λ_*ct*_ are cohort-by-year fixed effects; *ω*_*ic*_ are journal-by-cohort fixed effects; and *μ*_*ict*_ is a term of idiosyncratic errors. Cohort-by-year fixed effects serve a dual purpose in our model: they control for unobserved, time-varying differences across cohorts and, as a component in the difference-in-difference specification, serve as a post-intervention dummy for each cohort. Likewise, journal-by-cohort fixed effects control for unobserved, time-invariant difference across journals in different cohorts, while at the same time serving as a treatment dummy in each cohort. Our estimate of the treatment effect for multiple events is *δ*.

In addition to evaluating the treatment effect of OA for a “representative journal”, we further investigate the heterogeneous effect of OA on different types of journals. Journal characteristics (e.g., journal areas, publishers, and tiers) provide useful priori criteria for identifying journals that are likely to undergo different treatment effects. To this end, we split our dataset into subsamples by the abovementioned covariates and investigate the heterogeneous treatment effect of OA by comparing the coefficients from Eq 2 with these subsamples.

### Stratification-multilevel method

In the previous section, we investigated the heterogeneous treatment effect of OA with regard to journal characteristics by sample splitting based on predefined criteria. The splitting scheme set forth above can only provide us with insight into heterogeneity from a particular perspective. A researcher might then ask: is it possible to study the heterogeneous treatment effect on the basis of a full set of journal characteristics, considered as a whole? As we know, individuals differ not only in their separate characteristics, as depicted by covariates, but also in how they respond to a particular treatment. The likelihood of being treated, measured by a propensity score which summarizes all relevant information in the covariates, provides a useful solution. Following Xie et al. [43], we adopt the stratification-multilevel method and investigate the heterogeneous treatment effects of OA as a function of propensity score. This method also enables us to control for time-invariant journal characteristics in a cross-sectional specification.

In contrast to difference-in-difference, which corrects for bias by making assumptions about the performance of individuals of two groups before and after the treatment in the panel data, the stratification-multilevel method delivers an unbiased estimate by assuming unconfoundedness [49] (this assumption is also called “conditional independence”, or “selection on observables”) in cross-sectional data. The basic idea of this method is as follows: we categorize individuals into different strata based on their treatment propensity as estimated by probit regression. Treatment effects are then estimated for each stratum, and a linear trend is fitted across these strata to show the heterogeneous treatment effect. In our case, the analysis is performed in the following fashion:

1. We use probit regression to estimate the treatment propensity for all journals based on the full set of journal characteristics.
2. We construct balanced propensity score strata in such a way that there is no significant difference between the average values of covariates for the treatment and control groups. Most biases from observed confounders can be efficiently removed in this step.
3. We estimate (propensity score) stratum-specific treatment effects within each stratum using the following level-1 regression:
  $$\begin{array}{c} y_{ij}=\alpha_{j}+\gamma_{j}d_{ij}+\mu_{ij} \end{array}$$ (3)
  where *y*_*ij*_ is the CiteScore of journal *i* in propensity score stratum *j*, *α*_*j*_ is propensity score stratum *j*-specific fixed effect, *d*_*ij*_ is a dummy variable indicating whether or not a journal *i* in propensity score stratum *j* opens access, and *μ*_*ij*_ is the usual error term. *γ*_*j*_ is the slope that characterizes the estimated treatment effect within each stratum.
4. We estimate a linear trend across propensity score strata using variance-weighted least squares regression (level-2 regression), in an attempt to detect patterns of heterogeneous treatment effect:
  $$\begin{array}{c} \gamma_{j}=\rho +\phi j+\epsilon_{j} \end{array}$$ (4)
  where level-1 slopes *γ*_*j*_ are regressed on propensity score strata indexed by *j*, *ρ* is the level-2 intercept, *ϕ* is the linear trend across strata, and *ϵ*_*j*_ is the error term.

Since the abovementioned stratification-multilevel method can only be applied to cross-sectional data, we use the change in CiteScores *y*_*after*_ − *y*_*before*_ as the dependent variable. To be more specific, we calculate the change of CiteScore for each journal before and after the OA in each year as the outcome of interest. For OA events in different years, we simply pool the observations together. By doing this, we can investigate the heterogeneous treatment effect from the perspective of varying propensity to become open access.

## Results

### Open access effects

Table 3 provides the estimated effects of OA on CiteScores via the difference-in-difference method. Both overall effect of multiple events and effects in each year have been presented. The results in column 1 reveal an overall treatment effect of OA on CiteScore over five years. As we expected, becoming an open access journal will significantly improve the CiteScore of a journal by 0.147 on average. Column 2 through column 5 illustrate the estimated effects in each year as a single treatment. The effects of years 2011 and 2012 are significantly positive, with values 0.245 and 0.243 respectively. The effects of years 2013 and 2014 are not confirmed by our data, as indicated by insignificant coefficients. These results might be explained by the fact that there is an overall rising trend in journal CiteScores in our data from 2011 to 2014 as indicated by positive and significant trends over years. The journals treated in 2013 and 2014, because they have higher pre-treatment CiteScores in general, might be said to have less potential to improve their CiteScores by becoming open access. It is worth mentioning that the overall effect (0.147) estimated by Eq (1) is very close to the average of the four separate effects (0.142) estimated in each year by Eq 2.

**Table 3:** Main effects of journal open access on CiteScore.

Diff-in-diff	Multiple events (1)	2011 OA (2)	2012 OA (3)	2013 OA (4)	2014 OA (5)
Effect of OA	0.147*** (0.0384)	0.245*** (0.0875)	0.243*** (0.0723)	0.0444 (0.0768)	0.0355 (0.0605)
*trend*	0.0358*** (0.0029)	0.0352*** (0.0029)	0.0358*** (0.00216)	0.0332*** (0.00201)	0.0306*** (0.00254)
Constant	0.759*** (0.0141)	0.767*** (0.00287)	0.703*** (0.00672)	0.589*** (0.00713)	0.988*** (0.00883)
Observations	66,135	10,100	22,670	14,445	18,920
R^2^	0.031	0.048	0.036	0.033	0.022
Number of journals	13,227	2,020	4,534	2,889	3,784
Journal FE	Yes	Yes	Yes	Yes	Yes
Year FE	Yes	Yes	Yes	Yes	Yes
Cohort FE	Yes	No	No	No	No

Robust standard errors in parentheses

*** *p* < 0.01,

** *p* < 0.05,

* *p* < 0.1

**Fig 2:**
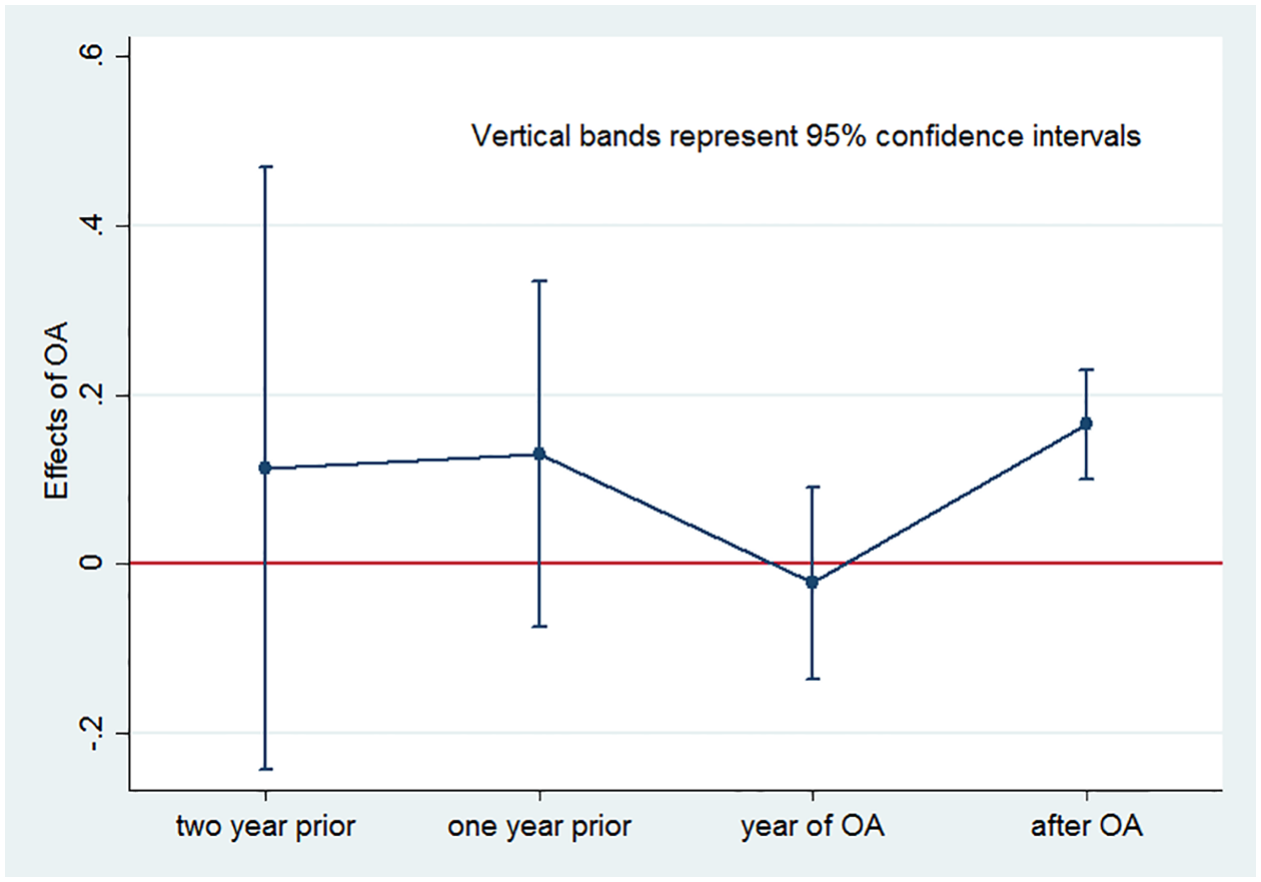
Effects of OA on CiteScore for years before and after the treatment.

As mentioned above, the assumption of parallel trend is critical for difference-in-difference to deliver an unbiased estimate. Our main results would thus be weakened if the trends in CiteScore of OA journals and non-OA journals are significantly different. Using a rigorous statistical test, we test this assumption for the periods before the treatment. In particular, we augment Eq 2 with the interaction terms of OA journal indicator with time dummies prior to the treatment as leads, in an attempt to check pre-treatment trends for the OA and non-OA journals. That is, we estimate:

$$\begin{array}{c} y_{ict}=\delta R_{ict}+\beta_{1}L_{ict}^{1}+\beta_{2}L_{ict}^{2}+\beta_{3}L_{ict}^{3}+\lambda_{ct}+\omega_{ic}+\mu_{ict} \end{array}$$ (5)

where *y*_*ict*_ is the CiteScore for journal *i*, treated in cohort *c*, in year *t*; *R*_*ict*_ is a dummy variable that equals 1 if journal *i* becomes open access by year *t* − 1 in cohort *c* and 0 otherwise; $L_{ict}^{1}$, $L_{ict}^{2}$, and $L_{ict}^{3}$ are dummy variables that equals 1 if year *t* is one year, two years, and three years prior to the open access of journal *i* in cohort *c*, receptively, and 0 otherwise; λ_*ct*_ are cohort-by-year fixed effects; *ω*_*ic*_ are journal-by-cohort fixed effects; and *μ*_*ict*_ is a term of idiosyncratic errors. We would expect insignificance of *β*_1_, *β*_2_, and *β*_3_ if CiteScore trends between OA and non-OA journals are the same prior to the treatment. Table 4 reports the estimated overall effects of OA on CiteScores estimated by Eq 5. The results reveal a significant effect of OA on CiteScore with a value of 0.165, which is close to our estimate (0.147) from Table 3. The insignificance of coefficients for the leads of 1-3 year prior mitigates the concern that OA and non-OA journals have different trends in CiteScore. We further present in Fig 2 the plot of these coefficients. It shows no significant pattern in difference between OA and non-OA journals before the practice of open access. Taken together, parallel trend assumption is not violated and the use of difference-in-difference is appropriate in our study.

**Table 4:** Overall treatment effect of OA on CiteScore with leads.

Diff-in-diff	Coefficient	S.E
Effect of OA	0.165***	(0.0232)
1 year prior	-0.0228	(0.0409)
2 years prior	0.131	(0.0737)
3 years prior	0.112	(0.129)
Constant	0.854	(0.00657)
Observations	66,135	
*R*^2^	0.032	
Number of journals	13,227	
Journal FE	Yes	
Year FE	Yes	
Cohort FE	Yes	

Robust standard errors in parentheses

*** *p* < 0.01,

** *p* < 0.05,

* *p* < 0.1

In order to understand whether the significance of *δ* in Eq 2 captures the overall effect of OA (the alternative being that the observation was coincidental), we randomly pick journals to undergo a placebo treatment [50], which we term pseudo-OA. That is, we subject these journals to the same analysis as if they have received the OA treatment. This place-bo should, on average, have no impact on the journals’ CiteScores. Therefore, we can compare our estimate of the effects of OA from Table 3 to that of the placebo treatment. To be specific, we randomly allocate a number of non-OA journals to match that of the treatment group (i.e., actual OA journals) in each year. Eq 2 is then used to estimate the treatment effects of pseudo-OA. We repeat this process 2000 times to build a distribution of placebo treatment effects. A significant difference between the two estimators would alleviate the concern that we have observed the effect of OA by mere chance.

**Fig 3:**
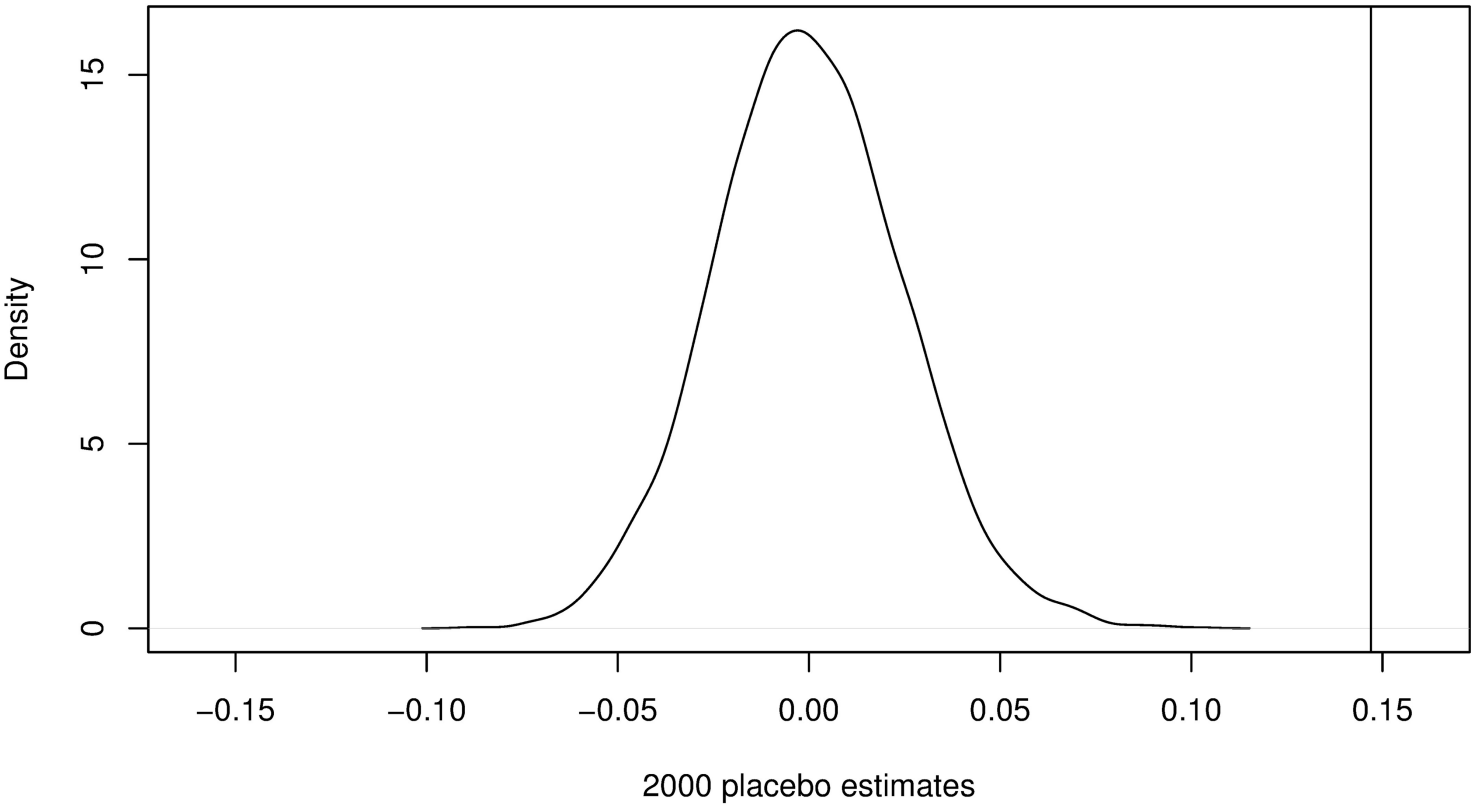
Density plot of the distribution of 2000 placebo estimates of the effect of OA on CiteScores.

Fig 3 plots kernel density estimates of the placebo treatment effects of pseudo-OA. The mean value is approximately zero, which verifies that there is no net effect of pseudo-OA on CiteScore. The vertical line on the right of Fig 3 represents the effect estimate (0.147) that we actually observed in our data. The observed effect falls in the right tail of the placebo treatment effects, which suggests that it is unlikely to have been observed due to chance.

### Heterogeneous treatment effect by journal characteristics

Our next objective is to estimate heterogeneous effects of OA based on journal characteristics by restricting our regression analysis over subsamples determined by Publisher, Area, and Rank.

We first divide the journals in our dataset by publisher. We classify Springer, Sage, Elsevier, Wiley-Blackwell, and Taylor & Francis as the Big Five publishers [38], since they are the institutions with established reputations. We are interested in whether the effect of OA on journals from Big Five publishers differs from the effect on journals from other publishers. Table 5 reports the results for the difference-in-difference estimate with the two subsamples.

**Table 5:** Heterogeneous effects of journal open access on CiteScore, by publisher.

Diff-in-diff	Big five publishers (1)	Other publishers (2)
Effect of OA	0.309*** (0.1075)	0.0742*** (0.0223)
*trend*	0.0483*** (0.0059)	0.0290*** (0.0032)
Constant	1.202*** (0.0280)	0.555*** (0.0152)
Observations	21,155	44,980
R^2^	0.061	0.020
Number of journals	4,231	8,996
Number of switches	78	166
Percentage of switches	1.84%	1.85%
Journal FE	Yes	Yes
Year FE	Yes	Yes
Cohort FE	Yes	Yes

Robust standard errors in parentheses

*** *p* < 0.01,

** *p* < 0.05,

* *p* < 0.1

The average effect of OA on the journals from Big Five publishers is 0.309, whereas the effect on the journals from other publishers is 0.0742. The two effects are both significant at very high confidence levels. However, it is the difference in estimated coefficients across different subsamples that we wish to emphasize. The t-statistic is 2.77 under the null hypothesis that the effect of OA is the same for the journals from the Big Five publishers and other publishers. This significant difference means that, if other journal characteristics are not accounted for, journals from Big Five publishers will benefit more when opening access. This result is easy to interpret because the quality of Big Five journals is usually guaranteed by their professional peer-review process, which would in turn attract more researchers after OA and boost their CiteScores.

We then investigate the effect of OA across different research areas. Journals in our dataset are manually categorized into six broad domains: Biology, Engineering, Math & Computer science, Medicine, Science, and Social science. We run difference-in-difference regressions for each cor-responding subsample, in an attempt to estimate heterogeneous effects of OA by area. Table 6 presents results for each area. We can see strong evidence for the significance of positive effects for the journals in Biology, Medicine, and Science, where open access effect leads to an average score increase of 0.400, 0.191, and 0.105 respectively. However, the effect of OA is insignificant or barely significant for journals in Math & Computer science, Social science, and Engineering. Unsurprisingly, we find that journals in different disciplines face different treatment effects when becoming open access.

**Table 6:** Heterogeneous effects of journal open access on CiteScore, by area.

Diff-in-diff	Biology (1)	Engineering (2)	Math & CS (3)	Medicine (4)	Science (5)	Social science (6)
Effect of OA	0.400*** (0.0819)	-1.000 (0.103)	0.154 (0.316)	0.191*** (0.0696)	0.105** (0.0476)	0.0218 (0.0418)
*trend*	0.0440*** (0.0088)	0.0331*** (0.0053)	0.0199*** (0.0072)	0.0378*** (0.0116)	0.0358*** (0.0050)	0.0286*** (0.0045)
Constant	1.100*** (0.0394)	0.742*** (0.0243)	0.980*** (0.0369)	0.683*** (0.0572)	0.932*** (0.0237)	0.475*** (0.0217)
Observations	8,935	6,115	3,425	18,195	13,965	15,500
R^2^	0.038	0.065	0.032	0.014	0.049	0.060
Number of journals	1,787	1,223	685	3,639	2,793	3,100
Number of switches	49	25	12	53	56	49
Percentage of switches	2.74%	2.04%	1.75%	1.46%	2.01%	1.58%
Journal FE	Yes	Yes	Yes	Yes	Yes	Yes
Year FE	Yes	Yes	Yes	Yes	Yes	Yes
Cohort FE	Yes	Yes	Yes	Yes	Yes	Yes

Robust standard errors in parentheses

*** *p* < 0.01,

** *p* < 0.05,

* *p* < 0.1

We also investigate the effect of open access by journal rank. The journals in our dataset are now divided into ranked subsamples corresponding to the top 10%, Quartile 1, Quartile 2, Quartile 3, and Quartile 4. Table 7 summarizes the regression coefficients for these subsamples. There is a significant treatment effect of OA for journals ranked in Quartile 2, Quartile 3, and Quartile 4, with an average CiteScore growth of 0.206, 0.181, and 0.146 respectively. However, the effect of OA on top-10% and Quartile 1 journals is not significant. As we predicted by the long tail theory, high-ranking journals realize less benefit from open access because researchers will always cite such journals in their fields, regardless of their access policies. Lower-ranked journals, in contrast, have more potential for CiteScore growth.

**Table 7:** Heterogeneous effects of journal open access on CiteScore, by tier.

Diff-in-diff	Top 10% (1)	Quartile 1 (2)	Quartile 2 (3)	Quartile 3 (4)	Quartile 4 (5)
Effect of OA	0.0561 (0.3514)	0.0268 (0.1703)	0.206*** (0.0703)	0.181*** (0.0418)	0.146*** (0.0473)
*trend*	-0.0553 (0.0349)	0.0093 (0.0285)	0.0251*** (0.0060)	0.0294*** (0.0035)	0.0509*** (0.0053)
Constant	5.382*** (0.1792)	3.340*** (0.1427)	1.131*** (0.0279)	0.515*** (0.0169)	0.0483* (0.0250)
Observations	2,020	5,275	19,015	21,135	20,710
R^2^	0.023	0.011	0.036	0.076	0.090
Number of journals	404	1,055	3,803	4,227	4,142
Number of switches	18	41	66	78	59
Percentage of switches	4.46%	3.89%	1.74%	1.85%	1.42%
Journal FE	Yes	Yes	Yes	Yes	Yes
Year FE	Yes	Yes	Yes	Yes	Yes
Cohort FE	Yes	Yes	Yes	Yes	Yes

Robust standard errors in parentheses

*** *p* < 0.01,

** *p* < 0.05,

* *p* < 0.1

### Heterogeneous treatment effect on propensity

Finally, we estimate the heterogeneous effects of OA by propensity score strata. In Table 8, we summarize the results of probit regression predicting the likelihood of becoming OA on the basis of journal characteristics. Table 8 suggests that Quartile 3 journals and Quartile 4 journals are more likely to open access, as are journals from non-Big Five publishers and journals with higher pre-treatment CiteScores. There seems, however, to be no pattern of preference with respect to a journal’s subject area or the year in which it opens access.

**Table 8:** Propensity score probit regression model predicting journal OA.

Probit regression	Coefficient	S.E
Quartile 4	0.231**	(0.0945)
Quartile 3	0.125*	(0.0732)
Quartile 1	0.130	(0.106)
Big five	-0.177***	(0.0621)
Year of OA	0.00369	(0.0254)
Biology	0.00029	(0.108)
Math & CS	-0.126	(0.146)
Medicine	-0.109	(0.100)
Science	-0.0587	(0.102)
Social science	-0.0286	(0.104)
Pre-OA CiteScore	0.234***	(0.0428)
Constant	-9.377	(51.20)
Observations	12,816	
*P* > *χ*^2^	0.000	

Standard errors in parentheses

*** *p* < 0.01,

** *p* < 0.05,

* *p* < 0.1

**Fig 4:**
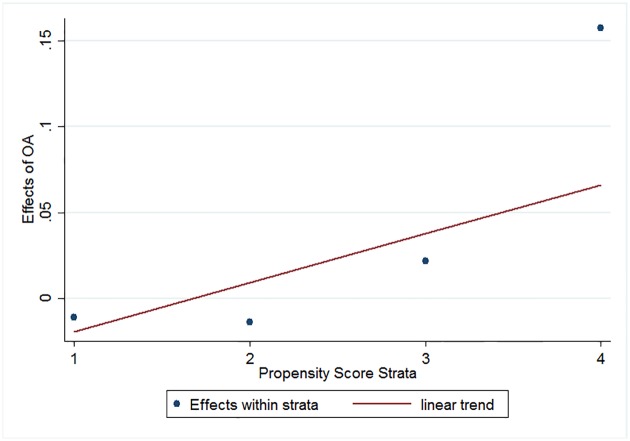
Effect of OA on CiteScore by propensity score stratum.

Fig 4 and Table 9 present the pattern of heterogeneous effects of open access by propensity score strata. The level-2 slope indicates a significant increase in the effect of OA, a difference of 0.03 for each unit change in propensity score rank. Among the level-1 slopes, which are the estimated treatment effect in each propensity stratum, the estimate for the journals from stratum 4 is significant. That is, on average, OA increases the CiteScore of journals that are most likely to open access by 0.143. This value is comparable to 0.147, the overall treatment effect estimated by difference-in-difference (Table 3). We can interpret the results of this of heterogeneous treatment effect on propensity as a mechanism at work: when journals with a high propensity for OA actually open access, they benefit most from doing so. On the other hand, for journals with a lower propensity, opening access may not be an effective strategy for improving CiteScore. Our findings are consistent with the positive selection hypothesis in sociology [51, 52]: the “promising” journals, in the sense of having more potential in the growth of their CiteScores, are most inclined to open access. We can take a snapshot of these journals: although most of them are not ranked especially highly in their fields, nor published by the Big Five, they are quality publications as indicated by higher pre-OA CiteScores. In other words, journal quality is an important factor for OA to take effect and obscure journals might benefit from OA only if they are quality journals that have potential in the growth of their CiteScores.

**Table 9:** Heterogeneous effects of journal open access on CiteScore, by propensity.

	Effect of OA	S.E
**Level-1 slopes**		
P-score stratum 1 [0.000-0.013]	-0.0124	(0.0197)
P-score stratum 2 [0.013-0.019]	-0.0325	(0.0325)
P-score stratum 3 [0.019-0.025]	0.0080	(0.0291)
P-score stratum 4 [0.025-1.000]	0.143**	(0.0589)
Observations	12,816	
**Level-2 slopes**		
Slope	0.0256*	(0.0145)
Constant	-0.0494	(0.0301)

Note: Propensity score strata were balanced such that mean values of covariates did not significantly differ between OA journals and non OA journals.

Standard errors in parentheses

*** *p* < 0.01,

** *p* < 0.05,

* *p* < 0.1

## Robustness check

In the results section, we studied the stability of difference-in-difference estimates over a variety of subsamples and derived quite consistent results. In this section, we test for the robustness of stratification-multilevel method by relaxing the unconfoundedness assumption. In most previous literature in social science and economics, unconfoundedness is assumed in order to investigate the treatment effect [53–55]. However, as Breen [56] has argued, the existence of unobserved confounders, if not accounted for, will nonetheless invalidate the assumption and introduce biases in analysis. Therefore, a test has been proposed: make adjustments to the outcome of interest when estimating the treatment effect to assess the results’ sensitivity to different types and degrees of bias. Adopting the notation of the potential outcomes approach [57], we denote *Y* as an outcome of interest with two potential outcomes for each individual (*Y*^1^, *Y*^0^), where *Y*^1^ is the potential outcome if an individual is treated, and *Y*^0^ is the potential outcome without treatment. Furthermore, we define a binary variable *D* indicating the treatment status, with *D* = 1 if an individual actually received treatment and *D* = 0 otherwise. The average treatment effect can be arranged as follows:

$$\begin{array}{c} ATE=\left[E\left(Y^{1}|D=1,X\right)-P\left(D=1|X\right)c_{0}\right]-\left[E\left(Y^{0}|D=0,X\right)+P\left(D=0|X\right)c_{1}\right] \end{array}$$ (6)

where

$$\begin{array}{c} c_{0}=E\left(Y^{0}|D=1,X\right)-E\left(Y^{0}|D=0,X\right) \end{array}$$ (7)

$$\begin{array}{c} c_{1}=E\left(Y^{1}|D=1,X\right)-E\left(Y^{1}|D=0,X\right) \end{array}$$ (8)

According to Eq 6, the biases are proportional to *c*_0_ or *c*_1_. We can thus correct for the biases by subtracting the term as a function of *c*_0_ or *c*_1_ from the outcome *Y*, provided that we can attain a reasonable calibration of *c*_0_ and *c*_1_. The adjusted outcome Y* can be computed as follows:

$$Y^{*}=\{\begin{array}{cc} Y-c_{1}P(D=0|X) & \text{if}\;D=1\\ Y-c_{0}P(D=1|X) & \text{if}\;D=0. \end{array}$$ (9)

The terms *P*(*D* = 0|*X*) and *P*(*D* = 1|*X*) can be estimated using probit regression. To calibrate *c*_0_ and *c*_1_, we follow Breen [56] and adopt a symmetric setting:

$$\begin{array}{c} c_{0}=c_{1}=\alpha \end{array}$$ (10)

where *α* is a non-negative constant. This corresponds to a positive pre-treatment bias: the average potential growth of CiteScores of the OA journals, had they not opened access, would have been greater than that of the non-OA journals. We calibrate *α* by changing its value in estimating the treatment effect where confounders are not conditioned on until it yields an estimate equal to the treatment effect conditioning on the observed covariates. Specifically, we first estimate the treatment effect of OA on *Y* using propensity score matching (PSM), a non-parametric causal model which assumes unconfoundedness, and obtain a result. Afterwards, we search for different values of *α* to estimate the treatment effect of OA on Y* using naïve estimator *E*(Y*|*D* = 1) − *E*(Y*|*D* = 0) until the result matches the one derived by PSM. Given the calibrated value of bias, denoted as $\hat{\alpha }$, we can now test how our estimator deviates from the original result with changing values of $\hat{\alpha }$ (e.g., $\frac{\hat{\alpha }}{2}$, $\hat{\alpha }$, $2\hat{\alpha }$, and $5\hat{\alpha }$).

As Table 10 shows, our estimates of heterogeneous treatment effect of OA by propensity score are very robust to this test: correction for the positive pre-treatment bias does not change the upward slope of CiteScore by OA propensity strata, which indicates a positive selection mechanism. This addresses our concern that the growth of CiteScore after OA might be attributed to the pre-treatment conditions of journals instead of OA itself.

**Table 10:** Robust analysis of heterogeneous effects of journal open access on CiteScore.

	*no bias* (1)	$bias=\frac{\hat{\alpha }}{2}$ (2)	$bias=\hat{\alpha }$ (3)	$bias=2\hat{\alpha }$ (4)	$bias=5\hat{\alpha }$ (5)
**Level-1 slopes**					
P-score stratum 1	-0.0124 (0.0197)	-0.0136 (0.0201)	-0.0156 (0.0201)	-0.0197 (0.0201)	-0.0317 (0.0201)
P-score stratum 2	-0.0325 (0.0325)	-0.0342 (0.0325)	-0.0362 (0.0325)	-0.0402 (0.0325)	-0.0522 (0.0325)
P-score stratum 3	0.0080 (0.0291)	0.0064 (0.0291)	0.0044 (0.0291)	0.0004 (0.0291)	-0.0116 (0.0291)
P-score stratum 4	0.143** (0.0589)	0.141** (0.0584)	0.139** (0.0584)	0.135** (0.0584)	0.123** (0.0584)
Observations	12,816	12,816	12,816	12,816	12,816
**Level-2 slopes**					
Slope	0.0256* (0.0145)	0.0257* (0.0145)	0.0257* (0.0145)	0.0257* (0.0145)	0.0257* (0.0145)
Constant	-0.0494 (0.0301)	-0.0512 (0.0305)	-0.0532* (0.0305)	-0.0571* (0.0305)	-0.0691** (0.0305)

Note: By introducing selection bias *α* in our model, we alter the assumption of unconfoundedness to check the robustness of our model. We calibrate *α* by changing its value in estimating treatment effect where confounders are not conditioned on until it yields an estimate equal to the treatment effect conditioning on the observed covariates.

Standard errors in parentheses

*** *p* < 0.01,

** *p* < 0.05,

* *p* < 0.1

In addition, we also empirically investigate how robust our estimates are when we exclude non-OA journals that contain a lot of papers supported by NIH, which requires funded papers published as open access. Specifically, we built a web crawler and simple calculator that can count the number of OA articles made available by NIH in each non-OA journal. We parse the number of articles available through PubMed Central (PMC) Citation Search for each non-OA journal in the control group. We find that only as low as 3% of journals have an average of 30 or more OA articles supported by NIH. As such, the impact of current funding policy on our results should be trivial. We further limit our sample to exclude the Non-OA journals with an average of 30 or more articles over 4 years on PMC from our sample. There are totally 386 instances of journals excluded. We redo our analyses based on the new sample and find our results barely change.

## Conclusions and discussion

This paper empirically investigates the open access effect on the CiteScore of journals. Utilizing a unique dataset and the difference-in-difference econometric technique, we were able to identify the potential causal effect of open access on journal CiteScores. We have found a positive effect for OA journals in general. However, the effect is more pronounced in journals that are published by the Big Five publishers, and in journals in Biology, Medicine and Science. More surprisingly, the OA effect is more pronounced in lower ranked journals than in high-ranking journals, suggesting a “long tail” effect. Besides, when considering the propensity of a journal to become OA, we found that the journals more likely to become OA derive a greater benefit when they actually become OA. This is consistent with the positive selection hypothesis in sociology. Furthermore, we reconcile the conflicting findings from previous studies regarding “long tail” vs. “superstar” effect by taking account for journal quality, and find obscure journals might benefit from OA only if they are quality journals that have potential in the growth of their CiteScores.

Implicitly, we assume that the journals transition from purely closed access to fully open access. However, there are journals that are closed access, yet contain articles which elect to be open access. This hybrid OA situation has the potential to greatly complicate an analysis of the OA effect. To discuss how this hybrid OA journals would affect the results of this study, we can consider three possible scenarios: 1) hybrid journals for OA group only, 2) hybrid journals for non-OA group and 3) hybrid journals for both OA and non-OA groups. If hybrid journals exist only within the OA group before they become OA, then the OA effect on journal citations may be underestimated. In practice, this is unlikely to be the case. For 2) and 3), if we are willing to assume that the elective OA articles are consistent in their percentage and their citations over the years of our sampling period, then our results will remain consistent, since neither of these two scenarios violates the parallel-trends assumption of the difference-in-difference method.

Our paper confirms previous studies on open access that suggest OA increase journal citations [9, 15, 19, 22, 27–29]. More importantly, our results have significant managerial implications to stakeholders of journals such as editors, publishers, authors, and readers when considering the open access decision of a journal. The magnitude of the increase in citations of a journal shall depend on characteristics of the journal such as the field, rank, and discipline of the journal, as well as the tendency of similar journals prone to open access. Moreover, very recently, libraries, universities and academic institutions in some countries, in forming a group, are trying to negotiate a deal with major publishers about open access (see source at http://www.sciencemag.org/news/2017/08/bold-open-access-push-germany-could-change-future-academic-publishing). The key issue that hinders reaching the deal is on how to set the price for open access. As the negotiation is becoming increasingly a hot debate, the heterogeneous effect of open access found in our study may help the publishers and authors/readers better negotiate the price in settling the deal. For example, instead of bargaining on a uniform price for all articles, the price can vary for articles of different journals based on the current and potential citation increase after OA.

The potential caveat of this research could be due to the addition of new journals to Scopus as researchers found that for a given topic or domain, the number of publications, sources, and citations typically have an upward trend (e.g., [58]; [59]) and proper normalization is sometime warranted to better make sense of diachronical data. As a multidisciplinary study, our research has a limited capacity to identify the discipline-specific activities of importance that may significantly influence CiteScores across different fields. Instead, we carefully examined the possible impact of the increasing number of journals. Specifically, the number of new sources between 2011 and 2015 and found only 2% of sources are new whereas the other 98% are consistently included in our dataset for the same time frame. At the domain level, the share of new journals ranges from 0.58% in medicine to 2.14% in biology. Given these small shares, we believe the impact of new sources on the statistical analysis and results should be limited. However, for the benefit of individual disciplines, future research can focus on the impact of the discipline-specific major changes such as the addition of new journals and new datasets.

For other future research, one can extend our current research to a couple of different directions. First, interdisciplinary fields would be interesting to study. Researchers can focus on interdisciplinary journals (e.g. data science journals) and investigate the cross-fertilization effects of OA. For example, how does OA in disciplinary journals (e.g. computer science, mathematics, and information science) impact interdisciplinary journal performances. Second, researchers can investigate the impact of OA from alternative perspectives other than citation advantage. For example, one can investigate the causal effect of OA on structural influence of journals [60] as measured by social network-based methods.
